# The Effects of Practice on the Concurrent Performance of a Speech and Postural Task in Persons with Parkinson Disease and Healthy Controls

**DOI:** 10.1155/2013/987621

**Published:** 2013-06-11

**Authors:** K. Bo Foreman, Stuart Sondrup, Christopher Dromey, Eon Jarvis, Shawn Nissen, Leland E. Dibble

**Affiliations:** ^1^University of Utah, Department of Physical Therapy, 520 Wakara Way, Salt Lake City, UT 84108, USA; ^2^Brigham Young University, Department of Communication Disorders, 120 MCKB, Provo, UT 84602, USA

## Abstract

*Purpose*. Persons with Parkinson disease (PD) demonstrate deficits in motor learning as well as bidirectional interference (the performance of one task concurrently interferes with the performance of another task) during dual-task performance. Few studies have examined the practice dosages necessary for behavioral change in rehabilitation relevant tasks. Therefore, to compare the effects of age and PD on motor learning during dual-task performance, this pilot study examined persons with PD as well as neurologically healthy participants during concurrent performance of postural and speaking tasks. *Methods*. Seven persons with PD and 7 healthy age-matched and 10 healthy young control subjects were tested in a motion capture facility. Task performances were performed concurrently and recorded during 3 time periods (acquisition (beginning and ending), 48-hour retention, and 1-week retention). Postural control and speech articulatory acoustic variables were measured. *Results*. Healthy young participants consistently performed better than other groups on all measured postural and speech variables. Healthy young participants showed decreased variability at retention, while persons with PD and healthy age-matched controls were unable to consistently improve their performance as a result of practice. No changes were noted in the speech variables. *Conclusion*. The lack of consistent changes in motor performance in any of the tasks, except in the healthy young group, suggests a decreased efficiency of motor learning in the age-matched and PD groups and argues for increased practice dosages during balance training.

## 1. Introduction

Parkinson disease (PD) is thought to begin in the peripheral nervous system and progress to the central nervous system through the enteric, autonomic, and olfactory pathways [1]. Only with neuronal cell loss in the midbrain does PD begin to manifest its cardinal motor signs (akinesia, bradykinesia, tremor, rigidity, and postural instability). Although these motor signs are the most recognizable features of PD, the neurology community is developing a greater appreciation of deficits that extend beyond motor function [2].

Two signs of PD that may have profound implications for rehabilitation potential are the impairments of motor learning and difficulty with performance of concurrent motor tasks (dual-task deficits) [3, 4]. Previous research suggests that persons with PD can demonstrate retention of practiced tasks (defined as learning) [5]. However, the retention is generally not as good as the retention for persons without the disease, and the overall rate of skill acquisition is slowed [6].

 One cause for disappointing intervention effects in neurologic rehabilitation may be the lack of appropriate dosage [7]. In support of this idea, studies that examined increased practice of single task activities such as balance reactions and volitional weight shifts in persons with PD have demonstrated improved center of mass control and protective stepping responses [8, 9]. Although persons with PD appear to benefit from single-task practice, less is known about their ability to benefit from practice in dual-task conditions. Dual task conditions acutely degrade postural performance [10–12] as well as gait steadiness and symmetry [13, 14] in individuals with PD. 

While a recent study demonstrated that persons with PD could benefit from dual-task practice during gait [5], to our knowledge, no studies have examined changes in measures of anticipatory postural control and stability as a result of practice in dual-task conditions. In addition, it is unclear if persons with PD can improve performance and retain these changes over time when exposed to dosages of practice commonly utilized in the clinic [15, 16]. 

To address this issue, this study examined the effects of age and PD on practice-based changes in concurrent postural task and speech motor task performance (dual-task condition). The postural control and speech tasks were chosen because of their limited response to pharmacological treatment [17, 18]. Given the limitations of pharmacological treatments, behavioural interventions such as practice may be the most promising means of improving postural control and speech in individuals with PD. Specifically, based on previous research [11, 13], we hypothesized that persons with PD would demonstrate performance deficits in concurrently performed postural and speaking tasks when compared to healthy age-matched and healthy young controls. In addition, based on a research examining motor learning in PD [6], we hypothesized that the effects of practice on concurrently performed postural and speaking tasks would be different for individuals with PD compared to neurologically healthy individuals. 

## 2. Methods

### 2.1. Design and Selection of Participants

This study included 3 groups: (1) persons with PD (PD group), (2) healthy age-matched control participants (AM group), and (3) healthy young adult control participants (HY group). Utilizing movement velocity outcomes for between group effect sizes from Dromey et al. [11] (Cohen's *d* = 1.6) and Jessop et al. [8] (Cohen's *d* = 1.4) a priori power calculations indicated that between 7 and 10 individuals in each group were needed to provide 80% power with an alpha level of 0.05. The accessible population for the PD group was individuals from our facility's Movement Disorders clinic. Inclusion criteria for the PD group included a confirmed diagnosis of idiopathic PD and mild to moderate disease severity (Hoehn and Yahr Stage I–III). For the HY group, participants had to be between 18 and 40 years of age. All three groups had to have the physical and cognitive abilities to actively participate in the study procedures. Exclusion criteria included individuals who were cognitively unable to understand or follow study instructions, previous surgical management of PD, or individuals with significant orthopaedic (i.e., fracture, moderate-to-severe osteoarthritis) or neurological (i.e., stroke, traumatic brain injury, and neuropathy) injury. The accessible population for the AM and HY groups was from the university community or relatives of the individuals with PD. A general exclusion criterion for all groups was a history of concomitant medical conditions that limited their ability to perform the proposed testing. 

### 2.2. Tasks, Instrumentation, and Procedures

 All subjects signed an IRB-approved consent form prior to participating. The postural control task was a stationary base of support rise to toes (RTT) movement during which participants were instructed to the following “rise to your toes as fast as you can and stay as high and stable as you can for 5 seconds.” This task was selected because it required participants to voluntarily move from a stable (full foot-to-ground contact) to an unstable posture (only forefoot-to-ground contact) and maintain a stable position. The RTT movement has been used as a measure of postural control in previous studies [11, 17]. The speech task involved the repetitive reading of standardized sentences that were selected because they allowed inferences about lingual excursions [11, 19, 20]. All testing was performed in the Motion Capture Core Facility using an 8-camera Vicon Motion Analysis System (Vicon Motion Systems, Centennial, CO, USA) and an Advanced Medical Technologies Inc. force platform (OR6-7 series, AMTI, Watertown, MA, USA). Speaking tasks were recorded using a stereo headset with a microphone (Logitech, Inc., Newark, CA, USA) and a computer-based audio recording program (Audacity, version 1.3.5). For testing, participants wore black tight fitting clothing and no shoes. Passive reflective markers were placed on bony prominences utilizing a 15-segment full body standardized gait analysis marker set (Plug-In Gait marker set; Vicon Motion Systems, Centennial, CO) [21, 22]. Following marker placement, participants were asked to stand in a comfortable position on the force plate, which was covered with solid color butcher paper. To insure standardized foot position between test periods, each individual's feet were traced with a marking pen, and these tracings were used as the starting position for all trials. In addition, all subjects were instructed to begin trials with their arms positioned comfortably at their side. Motion capture data (marker trajectories and kinetic data) were synchronized using Vicon Nexus Software (Vicon Motion Systems, Centennial, CO, USA). 

Individual performance of the postural control and speech tasks was tested prior to the initial trials of dual-task practice. A substantial dual-task deficit in both posture and speech measures has been reported on previously [11]. For this study, the postural control and speech tasks were performed simultaneously and were synchronized using an auditory cue triggered from loading a second force plate that emitted a loud sound when loaded over 10 Newtons. This trigger placed a timestamp in both the auditory and motion capture data for synchronization purposes and signaled the subject to simultaneously perform the postural control and speech tasks. 

Testing was performed over three time periods utilizing a classic motor learning paradigm that used an acquisition phase with a larger number of practice trials separated in time from two retention phases with fewer trials [23, 24]. The first time period was the acquisition phase (day 1) in which participants completed 21 dual-task trials segregated into 7 blocks of 3 trials with 30 seconds rest between trials and 2 minutes rest between blocks. The second and third time periods were the 48-hour retention phase and the 1-week retention phase, respectively. For both retention phases, participants completed 9 dual-task trials segregated into 3 blocks of 3 trials with 30 seconds rest between trials and 2 minutes rest between blocks. Testing (including participant and laboratory setup) took approximately 45–60 minutes for the acquisition phase and 20–30 minutes for the retention phases. For each trial, data were collected from the time an auditory cue was given until after the movement task was completed. Participants were supervised closely during all trials in order to prevent falls. Data gathered during trials were stored on the laboratory computer for later analysis. In order to control for dopamine replacement medication effects, persons with PD were tested at the same time each day with testing of the PD subjects beginning approximately 1 hour after taking their medications. 

### 2.3. Data Processing and Analysis

 The independent variables used for analysis were group assignment (PD group, AM group, and HY group) and practice phase (beginning of acquisition, end of acquisition, 48-hour retention, and 1-week retention). The dependent variables used for data analyses for the postural task were related to motor planning, postural coordination, and postural stability. 

Reaction time (RT) reflected the time from the go signal until the onset of COP movement. Previous motor control research supports that the processing time taken from a go signal until movement onset reflects motor planning [23, 25]. Longer reaction times taken prior to the beginning of movement are considered to reflect increased motor planning demands. 

In order to visualize the biomechanical coordination of the center of pressure during movement from the foot flat to the on toes position, we chose two postural coordination variables (center of pressure velocity (COP Vel) as well as center of mass (COM) and center of pressure (COP) difference) [26]. Center of pressure velocity was calculated as the rate of change of the net center of pressure during the initial 0.25 seconds of anterior COP movement. Greater COP velocity was interpreted as improved postural coordination while reduced COP velocity was interpreted as bradykinetic postural coordination. Center of pressure-COM difference (COP-COM difference) was calculated as the maximal difference between the sagittal plane locations of the COP and the vertical projection of the COM onto the floor. Previous research has shown that persons with greater postural coordination allow a larger separation of the COP and COM positions during postural control tasks than less stable individuals [17, 27]. 

Once participants had reached the peak of their RTT position, they were asked to remain stable for 5 seconds. The variable selected to reflect postural stability in peak heel raise position was the vertical heel position coefficient of variation (HH CV). The HH CV was calculated by dividing the standard deviation of the heel position by the average heel position during the middle 3 seconds of the RTT task [26]. In the context of the task constraints to remain as “stable as possible,” increases in linear measures of variability such as the coefficient of variation reflect reduced stability [28]. 

The speaking task involved the production of two target sentences that were read from a sheet of paper at a comfortable rate and loudness. The sentences on the paper were printed using a large font and positioned on a stand at a comfortable reading position for each subject. These sentences were: “the boot on top is packed to keep” and “the boy gave a shout at the sight of the cake”. These sentences were selected because they included the corner vowels and several diphthongs that allowed inferences about lingual excursions via measurement of the first and second formants. The speech-specific dependent variables were articulatory acoustic measures that reflected movements of the tongue [29]. A diphthong is sometimes called a *gliding vowel* because it is a combination of two adjacent vowels (as in *boy*). Diphthong duration was chosen to reflect diphthong transition time in msec, which is a measure of how long it takes to transition from the first to the second vowel. Formants are prominent acoustic features in the speech signal that change in frequency as the tongue moves during diphthong articulation. The first and second formant (F1 or F2) frequency change during the diphthong (transition extent in Hz) was chosen to reflect tongue displacement; therefore, lower frequency change reflected smaller articulatory excursions. The slopes of each formant transition (Hz/ms) were chosen to reflect tongue velocity; therefore, smaller slopes reflect lower velocities. The first formant reflected superior/inferior and the second formant reflected anterior/posterior movement of the tongue [29, 30]. The diphthong analyzed in the standardized sentences included /*ɔɪ*/ (boy). 

The recordings were analyzed with Praat software (version 5.0.47; Amsterdam, Netherlands). The methods for data reduction and analysis have been detailed previously [11]. The speech acoustic measures were selected because they have been associated in previous studies with changes in articulatory function in speakers with PD and have appeared to be sensitive to dual-task interference and practice-mediated improvements [11, 19, 20].

For each variable, the average of 3 consecutive trials (or one “block”) was used as the representative dependent variable. During the acquisition phase, the first block was used to represent the baseline, and the seventh block was used to represent the end of acquisition. In order to examine the participants' initial performance on the retention day, rather than have their performance be confounded by additional practice, we utilized the first block for analysis during both the 48-hour and one-week retention phases. 

Changes in performance were defined as changes that occurred during the acquisition phase, that is, between block 1 and block 7 of acquisition. Motor learning was defined as changes that occurred between the acquisition phase and the retention phases. Short-term learning was defined as the difference between block 1 of acquisition and block 1 of the 48-hour retention testing, while long-term learning was defined as the difference between block 1 of acquisition and block 1 of the one-week retention testing [23]. 

Because of the small sample size and the potential for violations of the assumptions of parametric statistical tests, we utilized nonparametric analyses. To examine between group differences at baseline, for each dependent variable we compared each group's acquisition block 1 performance using separate Kruskal-Wallis ANOVAs. Post hoc testing was performed as needed using Mann-Whitney *U* tests. To examine changes in performance during the acquisition phase and between acquisition and retention phases within groups, we compared acquisition block 1, acquisition block 7, 48-hour retention block 1, and 1-week retention block 1 using separate Friedman ANOVAs. Post hoc testing was performed as needed using Wilcoxon matched pairs tests. 

For all dependent variables, the magnitude of change was estimated by calculating the percent change from acquisition block 1. The level of significance for all comparisons was set at 0.05. All statistical analyses were performed with SPSS 19 (IBM Inc; Armonk, NY,USA) for Macintosh.

## 3. Results

Overall, 24 participants completed the study (7 persons with PD, 7 neurologically healthy age-matched controls, and 10 neurologically healthy young participants) (Table 1). All participants that were recruited completed all testing periods. 

### 3.1. Motor Planning

 Analysis of reaction time results showed no statistical differences between groups or over time (*P* > 0.05). The observed reaction times were consistent with those observed in other studies of dual-task paradigms [31] (Table 2).

### 3.2. Postural Coordination

COP velocity results for block 1 of the acquisition phase (baseline) demonstrated a significant difference between the groups (*P* = 0.006). Post hoc testing revealed that the HY group demonstrated significantly faster COP velocity at baseline relative to the AM and PD groups (*P* < 0.04). The PD group demonstrated the slowest velocity at baseline (Table 2).

Within-group analysis demonstrated that only the HY group showed a significant difference as a result of practice (comparison between acquisition and retention phases) (*P* = 0.02). Post hoc testing revealed that the HY group significantly increased their COP velocity from baseline to the end of the acquisition phase by 18.7% (*P* = 0.03). Center of pressure velocity for the AM and PD groups was not significantly altered at any phase of the study (Table 2).

 A significant difference was found between the groups in COP-COM difference for block 1 of the acquisition phase (baseline) (*P* = 0.003). Post hoc testing revealed the HY and AM groups demonstrated a significantly larger COP-COM difference relative to the PD group (*P* < 0.01). The PD group demonstrated the smallest COP-COM difference at baseline. 

Within group-analysis demonstrated that none of the groups showed significant differences in COP-COM difference as a result of practice (comparison between acquisition and retention phases) (Table 2).

### 3.3. Postural Stability

A significant difference was found between the groups in HH CV for block 1 of the acquisition phase (baseline) (*P* = 0.02). Post hoc testing revealed that the HY and AM groups demonstrated significantly less variability at baseline relative to the PD group (*P* < 0.02). The HY group demonstrated the lowest HH CV value, and the PD group had the highest HH CV at baseline (Table 2). 

Within-group analysis demonstrated a significant difference as a result of practice (comparison between acquisition and retention phases). Post hoc testing revealed that the greatest improvements in HH CV were seen in the HY group with significant decreases of 38% and 50% at 48-hour and one-week followups, respectively, relative to acquisition (*P* < 0.05). In contrast, there was no significant effect on HH CV in the AM or the PD groups. 

### 3.4. Articulatory Acoustic Measures

A significant difference was found between the groups for /*ɔɪ*/ F2 slope for block 1 of the acquisition phase (baseline) (*P* = 0.04). Post hoc testing revealed that the PD group produced significantly different values relative to the AM and HY groups (Table 3). 

Within-group analysis demonstrated no significant differences as a result of practice (comparison between acquisition and retention phases) in any of the three groups (*P* > 0.05) (Table 3).

## 4. Discussion 

Based on our interest in the effects of motor learning deficits on the ability to improve components of postural task performance in persons with PD, in this preliminary study we subjected persons with and without PD to commonly utilized practice dosage amounts [15] of dual-task practice. As we hypothesized, persons with PD demonstrated performance deficits in concurrently performed postural and speaking tasks when compared to healthy age-matched and healthy young controls. In addition, we hypothesized that persons with PD would respond differently to practice than neurologically healthy participants. Despite having the least variability in measures of postural stability, the healthy young participants were the only group that improved in HH CV during acquisition and retention. Such results suggest that the amount of practice necessary for postural task motor learning in healthy young individuals does not appear sufficient to drive changes in persons with PD or healthy elders. No consistent effect of practice was noted in any of the groups for the articulatory acoustic measures.

### 4.1. The Effects of Age and PD on the Efficiency of Acquisition and Retention

Neurologically healthy young participants consistently performed better on all measured postural and speech variables. As evidence of the adverse effect of age on the measured variables, most commonly the neurologically healthy age-matched control participants demonstrated impaired postural control and speech performance relative to the young participants. The effects of Parkinson's disease beyond age was specifically evident for our measures of COP velocity and heel height variability where participants in the PD group demonstrated substantially greater bradykinesia and variability than the neurologically health groups (COP velocity: PD = 155.49 mm/sec, AM = 247.84 mm/sec, HY = 369.31 mm/sec at block one of acquisition; heel height variability: PD = 26.40, AM = 6.80, HY = 4.20 at block one of acquisition). 

 In this study we examined a concurrently performed postural control task and a speech task using a classic motor learning paradigm [23, 24]. The postural control and speech tasks were chosen because of their limited response to pharmacological treatment [17, 18]. Given the limitations of pharmacological treatments, behavioural interventions such as practice may be the most promising means of improving postural control and speech in individuals with PD. However, since deficits in motor learning in individuals with PD or with basal ganglia lesions may be due to limited amount of practice [32], by limiting the number of practice trials, we introduced a commonly utilized practice dosage to the research design. We theorized that the bidirectional interference [11] that occurs between concurrently performed tasks would confound any differences in motor learning between the groups. The lack of consistent changes in motor performance in any of the postural tasks, except in the HY group, suggests a decreased efficiency of motor learning in the AM and PD groups and would suggest that additional amount of practice may be necessary. While previous research has utilized differing motor tasks and practice paradigms and typically examines the single-task performance [33–35], our results add evidence to the theory that a deficit in the retention of motor skill may necessitate additional amounts of practice [4, 36].

### 4.2. Task Difficulty

 Although the HY group demonstrated improvements in postural coordination and postural stability as a result of practice, consistent changes in speech task performance in any group as a result of practice were lacking. One potential reason for this was that the stereotyped speaking tasks that were used in this study utilized well-practiced words for primary English speakers. Because our measures of speech performance came from sentence production, the extensive familiarity the subjects had with these words likely limited our ability to observe any practice-related improvement [37]. Another potential reason for the lack of practice-related change in speech performance is that due to the acute consequences of falls, participants chose to prioritize postural stability over speaking. This lack of attention to a nonprimary task could have diminished any practice-related improvements. However, our previous research using these tasks suggests that in the absence of explicit instructions for prioritization, there appears to be bidirectional interference on both tasks [11]. That is, performance of both tasks at the same time resulted in concurrent performance deficits compared to individual performances of each task [38, 39].

### 4.3. Limitations and Directions for Future Research

 While these results suggest that practice dosages for motor learning may need to be different for persons with PD or advanced age, they should be interpreted with caution. Based on our research design, type I and type II statistical errors are possible. Because of the exploratory nature of this project, we did not perform corrections for multiple comparisons. Although our sample size was calculated using a priori power calculations, the effect sizes calculated from this data were smaller than those that we used and therefore we cannot ruleouttype II statistical errors. These smaller effect sizes suggest that dual tasking may reduce the practice effect on skill acquisition and imply that future studies will need to employ design features to address this effect (increased practice, larger samples). In addition, for the sake of internal validity, the tasks used were constrained measurements of postural and inferred vocal tract movement that were used for both practice and testing. Our selected speaking task may not have been challenging enough to reveal practice-mediated improvements in articulatory acoustic measures. Lastly, formal cognitive testing was not performed to assess learning abilities. Future research should include a larger number of subjects, utilize retention and transfer tasks, perform formal cognitive testing, and examine varied dosages of practice to elucidate the adequate dosage of practice to induce lasting changes in postural and speech task performance in both persons with PD and neurologically healthy elders. 

## 5. Conclusion 

 When asked to perform dual-task practice of postural motor and speaking tasks, persons with PD and neurologically healthy age-matched controls were unable to improve their performance during the acquisition phase or the retention phases. In contrast, when exposed to the same practice dosage, neurologically healthy young participants improved and retained improved postural stability over a one-week period.

## Figures and Tables

**Table 1:** Participant demographics.

	Groups		
Variable	HY group	AM group	PD group
n (gender)	10 (M = 4; F = 6)	7 (M = 5; F = 2)	7 (M = 7; F = 0)
Age	25.50 (2.40)	70.50 (11.90)	68.70 (9.20)
Time since diagnosis			4.11 (2.31)
Disease severity* (modified HY)			2 (1.5–3)
Taking carbidopa/levodopa			Yes: 6 No: 1
Dysarthria severity (1–10)*			4.1 (1–7)
6-month fall history			>2 falls: 3 0–2 falls: 4

HY group: healthy young adult controls; AM group: healthy age-matched controls; PD group: persons with PD.

*Median (range).

**Table 2:** Values (SD) for each postural control measure at acquisition and retention phases for each group.

	Acquisition (baseline)	Acquisition (ending)	Retention 48 hrs	Retention 1 week
	Block 1	Block 7	Block 1	Block 1
	mean (SD)	mean (SD)	mean (SD)	mean (SD)
Reaction time (sec)				
HY group	0.66 (0.16)	0.59 (0.14)	0.61 (0.10)	0.67 (0.11)
AM group	0.76 (0.21)	0.74 (0.36)	0.70 (0.25)	0.75 (0.29)
PD group	0.79 (0.20)	0.67 (0.36)	0.78 (0.22)	0.73 (0.10)
COP Vel (mm/sec)				
HY group	381.39 (166.64)^∗,#^	473.26 (146.95)	446.85 (179.12)	503.16 (217.22)
AM group	263.30 (184.21)	227.08 (227.76)	331.05 (301.86)	238.58 (229.46)
PD group	97.13 (261.83)	113.45 (235.31)	108.02 (219.66)	139.39 (219.58)
COP_COM diff (mm)				
HY group	52.84 (21.69)*	62.74 (26.56)	59.97 (24.40)	62.65 (22.95)
AM group	41.18 (26.45)	40.58 (9.75)	44.88 (19.60)	43.73 (14.40)
PD group	18.43 (25.80)	23.40 (23.15)	27.72 (21.49)	31.23 (26.52)
Heel Height CV (%)				
HY group	3.86 (2.50)^∗,∗∗^	2.79 (4.10)	2.58 (1.70)	2.07 (3.30)
AM group	2.43 (4.70)	3.72 (8.80)	3.01 (22.50)	2.18 (13.80)
PD group	14.35 (5.08)	24.74 (43.30)	13.59 (43.90)	9.03 (27.50)

HY group: healthy young adult controls; AM group: healthy age-matched controls; PD group: persons with PD.

All values: median (interquartile range).

*Significant difference between groups.

**Significant difference between acquisition block 1 (baseline) and block 1 at 48 hr retention and block 1 at 1 wk retention.

^#^Significant difference between acquisition block 1 and 7.

**Table 3:** Articulatory acoustic measures for /*ɔɪ*/ diphthong at acquisition and retention phases for each group.

	Acquisition (Baseline)	Acquisition (Ending)	Retention 48 hrs	Retention 1 week
	Block 1	Block 7	Block 1	Block 1
	mean (SD)	mean (SD)	mean (SD)	mean (SD)
/*ɔɪ*/ duration (sec)				
HY group	0.11 (0.03)	0.09 (0.04)	0.10 (0.03)	0.10 (0.03)
AM group	0.11 (0.05)	0.11 (0.04)	0.11 (0.04)	0.11 (0.04)
PD group	0.12 (0.03)	0.11 (0.02)	0.12 (0.02)	0.12 (0.03)
/*ɔɪ*/ F1 ext (Hz)				
HY group	87.17 (60.83)	66.83 (84.08)	63.17 (44.42)	56.83 (77.75)
AM group	108.66 (66.39)	75.67 (45.25)	61.67 (77.00)	59.00 (68.50)
PD group	78.67 (80.83)	74.16 (48.33)	44.50 (56.12)	47.67 (24.67)
/*ɔɪ*/ F2 ext (Hz)				
HY group	1066.00 (404.83)	938.17 (331.63)	997.33 (332.00)	915.00 (404.08)
AM group	1062.67 (302.67)	966.33 (248.08)	967.33 (273.92)	914.67 (231.17)
PD group	859.67 (219.00)	807.67 (290.00)	864.67 (268.33)	810.00 (308.17)
/*ɔɪ*/ F1 slope (Hz/ms)				
HY group	−0.78 (0.46)	−0.80 (1.01)	−0.67 (0.52)	−0.65 (1.12)
AM group	−0.89 (1.34)	−0.70 (0.68)	−0.49 (1.20)	−0.76 (1.14)
PD group	−0.68 (0.96)	−0.70 (0.47)	−0.30 (0.70)	−0.39 (0.63)
/*ɔɪ*/ F2 slope (Hz/ms)				
HY group	9.72 (3.20)	9.96 (4.22)	10.77 (4.58)	9.65 (4.93)
AM group	11.19 (6.16)	9.64 (4.68)	10.13 (4.79)	10.96 (3.76)
PD group	7.11 (2.62)*	7.41 (2.67)	6.96 (1.04)	6.44 (3.46)

HY group: healthy young adult controls; AM group: healthy age-matched controls; PD group: persons with PD.

All values: median (interquartile range).

*Significant difference between groups.
